# Editorial: Diversity and molecular diagnostics of fungi and oomycetes in plants

**DOI:** 10.3389/fcimb.2023.1305306

**Published:** 2023-10-10

**Authors:** Tingyan Xu, Tingting Dai, Jing Si, Xin Li

**Affiliations:** ^1^ Co-Innovation Center for the Sustainable Forestry in Southern China, Nanjing Forestry University, Nanjing, Jiangsu, China; ^2^ Advanced Analysis and Testing Center, Nanjing Forestry University, Nanjing, Jiangsu, China; ^3^ Institute of Microbiology, School of Ecology and Nature Conservation, Beijing Forestry University, Beijing, China

**Keywords:** *Basidiomycota*, *Ascomycota*, taxonomy, phylogeny, ecological function, CRISPR-Cas12a

Fungal and oomycete plant pathogens are responsible for the devastation of various ecosystems such as forest and crop species worldwide. Fungi, as a separate kingdom, have the second largest number of species among eukaryotic taxa, while only 6.8% (Globally fungi about 2.2 million, reported 150,000) of the total number has been reported worldwide (Hawksworth & Lücking, 2017). Oomycetes are fungal-like organisms phylogenetically related to algae (Derevnina et al., 2016). Thus, there is still a large amount of fungal “dark matter” in the natural environment waiting to be discovered (Grossart et al., 2016). The discovery of new taxa of fungi and detection of provides a scientific basis for the rich diversity of organisms in nature and contributes to understand its ecological role in ecosystems (Hongsanan et al., 2023). The descriptions and documentation of these new taxa not only increase our knowledge of this group of organisms, but also provide important information for future research on fungi, such as better conservation and management of their unique ecosystems.

Eleven phyla of fungi have been reported (https://www.catalogueoflife.org/, retrieval on 25 Sept. 2023). The most described of these are Ascomycetes and Basidiomycetes fungi. Ascomycetes, which form ascospores, are different from Basidiomycetes composed of multicellular mycelia. There is no doubt that both play important ecological roles in various ecosystems. PCR and RPA-CRISPR/Cas12a detection methods play an increasingly important role in sensitive and accurate detection in various fields. Here, we proposed the Research Topic “Diversity and Molecular Diagnostics of Fungi and Oomycetes in Plants” which ended up with 17 articles from various geographic regions covering 29 new taxa of fungi and molecular detection of fungi and oomycetes.

Ascomycete are the most abundant in the fungal kingdom. The family *Herpotrichiellaceae* belongs to Ascomycota with setose, ostiolate ascomata, and dematiaceous hyphomycetes of its asexual morphs. They are distributed worldwide, including on insects, plants, rocks, and in the soil. Thitla et al. investigated rock of forests, and isolated 12 strains of rock-inhabiting fungi belong to *Herpotrichiellaceae*. Based on multi-gene phylogenetic analyses of a combination of ITS, LSU, SSU, TUB, and EF1A regions and morphological characteristics, a novel genus (*Petriomyces*) and three new species of *Cladophialophora* (*Cl*. *rupestricola*, *Cl*. *sribuabanensis*, and *Cl*. *thailandensis*) were described and illustrated. *Dothideomycetes* is the largest and most diverse class of Ascomycota. The class are commonly characterized by bitunicatea and fissitunicate asci. But most of the taxa lack molecular data and possess uncertain morphological features. There are still more than 200 genera that cannot be placed in any family or order. Therefore, further research is needed to determine its taxonomic status more precisely. Hongsanan et al. resolve ambiguous taxa in *Phaeothecoidiellaceae* and introduced two new genera (*Pseudorepetophragma* gen. nov. and *Pseudostomiopeltis* gen. nov.), one new species (*Pseudostomiopeltis xishuangbannaensis* gen. et sp. nov.), and three new combinations (*Pseudorepetophragma zygopetali* comb. nov., *Pseudostomiopeltis phyllanthi* comb. nov., and *Exopassalora sinensis* comb. nov.). Ascomycete are also considered to have important industrial applications, such as biocontrol agent for managing animal and plant pathogen. *Sarocladium terricola* has been recognized as a biocontrol agent for managing animal and plant pathogens. Not only that, study has shown that the fungus can also be used as an animal feed additive. Wang W. et al. sequenced, assembled, and annotated the genome of *S*. *terricola*. The phylogenomic results supported that *S*. *terricola* was closely related to the family *Bionectriaceae*. *Sarocladium terricola* cultured in a PDA medium had higher ergosterol content and relevant genes ERG3, ERG5, and ERG25 significantly up-regulated. This offered a scientific basis for the application of *S*. *terricola* as an animal feed additive. Basidiomycetes is another large group in the fungal kingdom. Most wood-inhabiting Basidiomycetes are pathogenic and can cause white or brown rot. *Hymenochaetaceae* has mainly been reported from tropics. Members of the genus *Fulvifomes* and *Pyrrhoderma* of *Hymenochaetaceae* are mostly on angiosperms and cause a white rot. Zhou M. et al. discovered two new species in their investigation on tropical Asian and American hymenochaetaceous fungi. Their taxonomic status was determined by morphological and phylogenetic analysis, and named *Fulvifomes acaciae* sp. nov. and *Pyrrhoderma nigra* sp. nov. They also provided an identification key to *Fulvifomes* and *Pyrrhoderma*. The genus *Leptoporus*, another wood-decaying polypore genus, can cause a brown rot on dead conifers and is mainly distributed in the North Hemisphere (North America, Europe, and Asia). Liu et al. reported one undescribed species of *Leptoporus* in the Hengduan Mountains of Southwest China. According to the molecular phylogenetic analyses of ITS + nLSU + RPB1 + RPB2 + TEF1 sequences and morphological characteristics, *L*. *submollis* was described and illustrated. Polyporales are accommodates massive corticioid fungi and cause a white rot on dead wood. Polyporales clade phlebioid, especially corticioid fungi, is mostly based solely on morphology and has not been intensively studied. Zhang Q-Y et al. confirmed their affinity using ITS and nLSU rDNA sequences. Both morphological characteristics and molecular evidence demonstrated that two new species belong to *Phanerochaete* and *Rhizochaete* named *P*. *shenghuaii* and *R*. *variegate*, respectively. The differences of phylogenetically related and morphologically similar species to the two new species were discussed. The results confirmed the existence of more unknown species in China. *Haploporus* is a cosmopolitan genus, belonging to the *Polyporales* also. It is characterized by resupinate to pileate basidiomata and can cause a white rot on wood with an important ecological function. Man et al. investigated the diversity of polypore and described two new species of *Haploporus* from Ecuador and China based on morphological characters and phylogenetic analyses of the ITS, LSU, and mtSSU sequences. The result shows that a high diversity of *Haploporus* exists in neotropical areas. They also provided an identification key to 25 known poroid *Haploporus* species. The genus *Rigidoporus* is an important parasite on cultivated tropical trees and causes a white rot. Wang C-G et al. made a comprehensive study regarding *Rigidoporus* displayed including morphological and phylogenetic analyses (using ITS + nLSU sequences). *Rigidoporus* including 18 species were divided into four clades. Three new species (*R*. *imbricatus*, *R*. *subcorticola*, and *R*. *pterocaryae*) and one new combination (*R*. *illavensis*) were described from China. They also provided the main morphological characteristics of the currently accepted species of *Rigidoporus*. Zhang Q-Y et al. investigated the diversity of wood-inhabiting fungi from China and reported two new species of *Scytinostroma acystidiatum* and *S*. *macrospermum* from Southwest China, based on ITS + nLSU dataset and morphological characteristics. They provided an identification key to the morphologically similar and phylogenetically related species known. The genus *Steccherinum* is wood-inhabiting fungi much known for its type *S*. *ochraceum*. Dong et al. explored the diversity and phylogeny of *Steccherinum* in China and described three new species (*S*. *fissurutum*, *S*. *punctatum*, and *S*. *subtropicum*). The genus *Fuscoporia* is a poroid, wood-decaying fungi. Chen et al. based on molecular genetic analyses and morphological criteria confirmed that the two new species belonging to *Fuscoporia* are described as *F*. *hawaiiana* and *F*. *minutissima*. They also provided an identification key to the North American species of *Fuscoporia*. Jelly fungi are a special group of wood-inhabiting Basidiomycetes and the diversity of the Chinese Jelly fungi is not well-known. The genus *Pseudohydnum* typified by *P. gelatinosum*, with gelatinous basidiomata with conical spines. Zhou H-M et al. collected some samples belonging to *Pseudohydnum* in the investigation of jelly fungi in North China, and found three unknown species. They determined the taxonomic status based on morphological evidence and phylogenetic analyses of ITS and LSU sequences datasets. They also provided a list about the main characteristics, type localities, and hosts of *Pseudohydnum* species. Basidiomycetes also include some ectomycorrhiza, which form reciprocal symbionts with plants, promote plant growth, improve host stress resistance, and play important ecological functions. The genus *Cortinarius* contains important ectomycorrhizal fungi but its diversity is poorly studied in China. Zhang Q-Y et al. described three new species of *Cortinarius* section *Anomali* from China based on morphological characters and phylogenetic analysis of the ITS sequences. These results contributed to enriching the diversity of Basidiomycetes worldwide.

Conventional PCR, triple PCR, Real-time fluorescence PCR, and Loop-mediated isothermal amplification (LAMP) were molecular detection methods currently in use for the detection of fungi and oomycetes in Plants. Wang D. et al. established a ddPCR method which enabled sensitive detection and accurate quantification of *V. nonalfalfae* and *V. alboatrum*, providing a valuable tool for evaluating disease progression and enacting effective disease control. Zhou H-M et al. provided a loop-mediated isothermal amplification (LAMP) method based on the glyceraldehyde 3-phosphate dehydrogenase (GAPDH) target for diagnosing root and butt rot caused by *Heterobasidion annosum* and surveillances in the ports of logs imported from Europe. Guo et al. developed a simple, rapid, sensitive, unaided-eye visualization, RPA-CRISPR/Cas12a-based detection system for the molecular identification of *P. ramorum* and *P. sojae* that does not require technical expertise or expensive ancillary equipment. All these present novel assays exhibit high specificity and sensitivity, and a short detection time. The method enables visualization of the results without the need for expensive equipment and facilitates the early detection of the pathogen. The assays were sensitive, efficient, and convenient. Practitioners could consider improvements to this assay to increase the sensitivity and expand detection to other pathogens.

## Author contributions

TX: Writing – original draft, Conceptualization, Investigation. TD: Writing – original draft, Writing – review & editing. JS: Formal Analysis, Writing – original draft, Writing – review & editing. XL: Conceptualization, Data curation, Methodology, Writing – original draft.
